# Editorial: Brain hypoxia and ischemia: New insights into neurodegeneration and neuroprotection, volume II

**DOI:** 10.3389/fnins.2022.1125883

**Published:** 2023-01-09

**Authors:** Natalia N. Nalivaeva, Elena A. Rybnikova

**Affiliations:** ^1^I.M. Sechenov Institute of Evolutionary Physiology and Biochemistry, Russian Academy of Sciences, Saint Petersburg, Russia; ^2^I.P. Pavlov Institute of Physiology, Russian Academy of Sciences, Saint Petersburg, Russia; ^3^School of Biomedical Sciences, Faculty of Biological Sciences, University of Leeds, Leeds, United Kingdom

**Keywords:** epilepsy, exosomes, glucocorticoids, ischemic stroke, neuroprotection, pre-conditioning, post-conditioning, prenatal hypoxia

Since the first issue of the Research Topic “*Brain hypoxia and ischemia: New insights into neurodegeneration and neuroprotection*” was released, numerous studies have expanded our understanding of the molecular mechanisms by which hypoxia can exert its destructive or protective actions. Normal brain development and function depend immensely on oxygen supply and its insufficiency, either due to reduced oxygen levels in the environment (hypoxia) or reduced blood flow (ischemia), can lead to neuronal cell death and subsequent neurodegeneration. Oxygen deficiency affects significantly various cellular functions starting with an immediate response on the composition of membrane lipids, changes in enzyme activity, mitochondrial remodeling, and subsequently resulting in changes in gene expression and translation. Impaired vascular health and reduced oxygen supply to the brain are linked to the pathogenesis of many neurodegenerative disorders including vascular dementia and Alzheimer's disease. Moreover, maternal hypoxia during pregnancy or fetal hypoxia/ischaemia in labor significantly affect new-born brain development and functions, thereby increasing the risk of developing various neurological pathologies in later life. Intensive research during the last decade has suggested various therapeutic avenues for developing treatments and preventive approaches against the pathological effects of hypoxic and ischemic insults which include hypoxic pre- and post-conditioning aimed at increasing brain hypoxic–ischaemic tolerance. In the recent years following the COVID-19 pandemic it became evident that hypoxia is one of the major factors which accompanies this systemic disease, leading to long-lasting neurological manifestations in patients not only with severe hypoxic conditions but also with milder symptoms. We were pleased with the number of papers submitted to the second issue of this Research Topic demonstrating the great interest of the research community in studying the effects of hypoxia on the brain starting from prenatal development up to the aging population and also reviewing various therapeutic avenues.

Using a model of acute prenatal hypoxia in rats in the period of embryogenesis when generation of the pyramidal cortical neurons takes place Amakhin et al. have demonstrated that hypoxia on the 14th day of embryogenesis mainly affected the progenitors of cortical glutamatergic neurons but not GABAergic interneurons or hippocampal neurons resulting in a reduced number of NeuN-positive neurons in the entorhinal cortex (EC) but not in the CA1 field of the hippocampus. However, the principal neuronal electrophysiological characteristics were altered both in the EC and hippocampus of animals exposed to hypoxia. Using the dosed electroshock paradigm, they found that seizure thresholds were lower in the hypoxic group making them more prone to developing seizures which suggests that prenatal hypoxia might be among the factors predisposing to epilepsy.

On the other hand Zhang et al. using magnetic resonance imaging have demonstrated that in a human population residing in chronic hypoxic environment at high altitude (4,300 m above sea level) there are significant changes in structural and physiological characteristics of the brain including decreased gray matter volume, damaged nerve fibers, and unbalanced intensity of neuronal activity in different brain regions compared to residents of lower (1,700 m) altitude. These alterations may underlie the structural and functional basis of lower scoring in cognitive tests observed in the high-altitude population due to chronic hypoxia. In line with these observations, the work by Ponirakis et al. reports the role of cerebral ischemia in development of mild cognitive impairment and dementia. It demonstrates that patients with subcortical and cortical ischemia had decreased hippocampal volume, corneal nerve fiber length and larger ventricular volume which correlated with cognitive impairment.

Several review artices in this Research Topic have critically assessed therapeutic strategies for treatments of the consequences of brain hypoxia and ischemia developed in recent years. In this regard Tang et al. have analyzed a possible role of the silent information regulator 1 (SIRT1) and its involvement in regulation of autophagy and attenuation of the cerebral ischemia-reperfusion outcome. The authors have discussed the molecular mechanisms by which both SIRT1 and autophagy exert their neuroprotective effects and their interaction, which provide a platform for developing drugs targeting SIRT1 levels in the brain. Another possible approach for treatment of patients with ischemic stroke (IS) is utilization of exosomes derived from mesenchymal stem cells as critically reviewed by Xiong et al. Due to the nano-scale of exosomes, their ability to cross the blood–brain barrier and low immunogenicity, they can be used as carriers of regulatory molecules (including microRNAs) aiming to improve the microenvironment of the ischemic tissue and regulation of neuronal activity. Although only applied in animal models, the method of transcranial direct current stimulation (tDCS) might also represent a neuroprotective approach in treatment of IS consequences as reviewed by Huang et al. Such treatments might have some beneficial effects reducing infarct size and improving neurological deficits, although the optimal application of tDCS and their full therapeutic potential require further investigation.

One of the important aspects in designing effective therapy for patients with brain IS is understanding their responsiveness to treatment which to a great extent depends on their hormonal status regulated by the hypothalamic-pituitary-adrenocortical (HPA) axis. As very convincingly presented by Gulyaeva et al. both clinical data and the results from rodent ischemia models show that glucocorticoids are involved in IS-induced brain dysfunction, in particular *via* glucocorticoid-dependent distant hippocampal damage. Designing valid models that reproduce the state of the HPA axis in clinical cases of IS will help both to plan better pre-clinical research and reinforce the diagnostic and prognostic potential of cortisol and other HPA axis hormones.

To stimulate brain tolerance to IS and also to enhance endurance and working capacity of the whole organism, various modes of hypoxic training have been developed over the years which are critically reviewed by Rybnikova et al. This review highlights the importance of studying molecular mechanisms by which intermittent hypoxic training mediates its neuroprotective effects at the tissue and molecular levels and emphasizes its high preventive potential against neurodegeneration and age-related cognitive decline.

## Author contributions

All authors listed have made a substantial, direct, and intellectual contribution to the work and approved it for publication.

